# The Disclosure of Bad News Over the Phone vs. in Person and its Association with Psychological Distress: a Systematic Review and Meta-Analysis

**DOI:** 10.1007/s11606-023-08323-z

**Published:** 2023-08-08

**Authors:** Jonas Mueller, Katharina Beck, Nina Loretz, Christoph Becker, Sebastian Gross, René Blatter, Tabita Urben, Simon A Amacher, Rainer Schaefert, Sabina Hunziker

**Affiliations:** 1 Department of Medical Communication and Psychosomatic Medicine, University Hospital Basel, Basel, Switzerland; 2 Emergency Department, University Hospital Basel, Basel, Switzerland; 3 Faculty of Medicine, University of Basel, Basel, Switzerland

**Keywords:** breaking bad news, disclosure, phone, anxiety, satisfaction

## Abstract

**Background:**

Communicating bad news such as a new cancer diagnosis to patients may have a major impact on their well-being. We investigated differences in patients’ psychological distress due to the disclosure of bad news by telephone compared to in person in a systematic review and meta-analysis.

**Methods:**

We included all studies that investigated anxiety, depressive or post-traumatic stress disorder (PTSD) symptoms in adult patients in whom bad news by telephone compared to in person were disclosed. We systematically searched PubMed, Embase, PsycINFO and CINAHL from the inception of each database to October 18, 2022. We included randomized and non-randomized trials.

**Results:**

We screened 5944 studies and included 11 studies in the qualitative analysis and 9 in the meta-analyses, including four randomized controlled trials. Overall, the quality of studies was moderate to good. There was no difference regarding psychological distress when bad news was disclosed by telephone compared to in person with similar symptom levels of anxiety (3 studies, 285 participants; standardized mean difference [SMD] 0.10 [95% CI -0.15 to 0.35]), depression (3 studies, 284 participants; SMD 0.10 [95% CI -0.30 to 0.49]), and PTSD (2 studies, 171 participants; SMD -0.01 [95% CI -0.48 to 0.36]). Results were similar for satisfaction with care.

**Discussion:**

This meta-analysis found no difference regarding psychological distress regardless if bad news were disclosed by telephone or in person, but there were overall only few and heterogeneous studies with a small number of eligible patients. The findings suggest that the modality of disclosure might play a secondary role and the way in which the bad news are communicated might be more important.

**Supplementary Information:**

The online version contains supplementary material available at 10.1007/s11606-023-08323-z.

## Introduction

Breaking bad news to a patient or a next of kin is a challenging conversation as the information disclosed often has an altering effect on the person’s life perspective. Herein, the way how bad news are communicated might play an important role for patients' psychological burden.^1–5^ Several communication techniques and guidelines such as specific communication protocols^6^ were developed, to facilitate the disclosure of bad news to patients and relatives.

For a long time, experts recommended disclosing bad news in person whenever possible^2, 3, 7^ as it renders addressing patients’ or relatives’ emotional responses more easily. Still, it was acknowledged that in certain situations it is more feasible to disclose bad news by telephone.^8^ Particularly, in case of a clinical deterioration or even sudden death of a patient, the imminent disclosure of the bad news over the phone might spare the next of kin a prolonged time of fearful uncertainty.^9, 10^ Around the year 2000, approximately one quarter of patient-physician conversations were conducted via telephone^11^ and with further development of mobile communication technologies and a growing need for cost-effective treatments, telephone consultations have become even more common. In fact, the current COVID-19 pandemic brought a sudden increase in telemedicine in order to minimize the risk of spreading the virus^12, 13^ and due to hospitals’ visitation restrictions. Since 2020, medical conversations via telephone including the disclosure of bad news are often a necessary substitute for in-person appointments and became an integral part of clinical practice across the world.

Therefore, further insight regarding the psychological impact of breaking bad news by telephone on patients and next of kin compared to breaking bad news in person is needed.

The aim of this systematic review and meta-analysis was to investigate whether disclosure of bad news by telephone is an appropriate alternative to in-person disclosure in terms of psychological distress and satisfaction with care measured by symptoms of anxiety, depression, and post-traumatic stress disorder (PTSD), as well as patient satisfaction.

## Methods

### Types of Studies, Participants, and Outcomes

We conducted this systematic review and meta-analysis in accordance with the updated version of the Preferred Reporting Items for Systematic Reviews and Meta-Analyses reporting guidelines (PRISMA 2020)^14^ and registered it in the International Prospective Register of Systematic Reviews (PROSPERO; ID: PROSPERO 2021 CRD42021233266). We included peer-reviewed observational studies, randomized controlled trials (RCT) and quasi-RCTs that investigated differences in psychological distress of breaking bad news by telephone compared to in person in patients or next of kin.

Our primary outcome was psychological distress defined as symptoms of anxiety, depression or PTSD. Our secondary outcomes were satisfaction with care including trust in the healthcare worker disclosing the bad news. Studies were eligible if they reported results on at least one of our primary or secondary outcomes. No restrictions concerning age or gender of adult participants and no publication date restrictions were applied.

Exclusion criteria were 1) no participants ≥ 18 years, 2) studies only including patients with psychiatric diagnoses, 3) studies only including patients with moderate to severe cognitive impairment, 4) no comparison of telephone versus in-person disclosure, 5) no results on at least one of the primary and secondary outcomes, and 6) conference articles or abstracts and case reports.

This manuscript is based on the MOOSE Checklist of Meta-analyses and Observational Studies.^15^

### Database Search for the Identification of Studies

We searched the digital databases PubMed, Embase, CINAHL and PsycInfo using a string of search terms consisting of subject headings and free-text words which we had developed together with an academic librarian experienced in systematic reviews (C.A.-H.). The search strings and filters for each database search can be found in the Supplementary Material. To identify additional studies, we screened all references of eligible studies through the cited references search of Web of Science and PubMed. The latest search was performed on October 18, 2022.

### Study Selection

Two investigators screened the titles and abstracts of articles regarding inclusion and exclusion criteria and independently assessed the full texts of all remaining studies. Disagreements were resolved through discussion with a third reviewer. Two investigators independently extracted the relevant data from the included studies.

### Risk of Bias Evaluation

We evaluated the risk of bias for every relevant outcome of all included studies using The Cochrane Collaboration’s tool for assessing risk of bias.^16^ Two authors independently assessed the risk of bias for all studies and resolved disagreements by discussion until consensus was found. A detailed description of the risk of bias assessment can be found in the Supplementary Material.

### Analysis

We synthesized the findings on primary and secondary outcomes of all studies in a qualitative analysis. Studies that provided data on the mean and standard deviation of psychometric scores assessing one of the outcomes and/or the numbers of patients with and without the outcome were included in the meta-analysis. We pooled continuous data using random-effects models and reported the standardized mean difference, i.e. inverse variance (IV) with 95% confidence intervals (CI). Heterogeneity was examined through visual inspection of the forest plots. We evaluated dichotomous data with a random-effects model applying the Mantel–Haenszel method and reported odds ratios (OR) and 95% confidence intervals. For the latter, we used the I^2^ statistic, which quantifies inconsistency across studies, to assess the consequences of heterogeneity on the meta-analysis. For all analyses, a two-sided *p*-value < 0.05 was considered statistically significant. Statistical analyses were conducted using the METAN package in Stata (Stata MP, version 15.1; StataCorp LP).

## Results

### Study Selection

**Figure 1:**
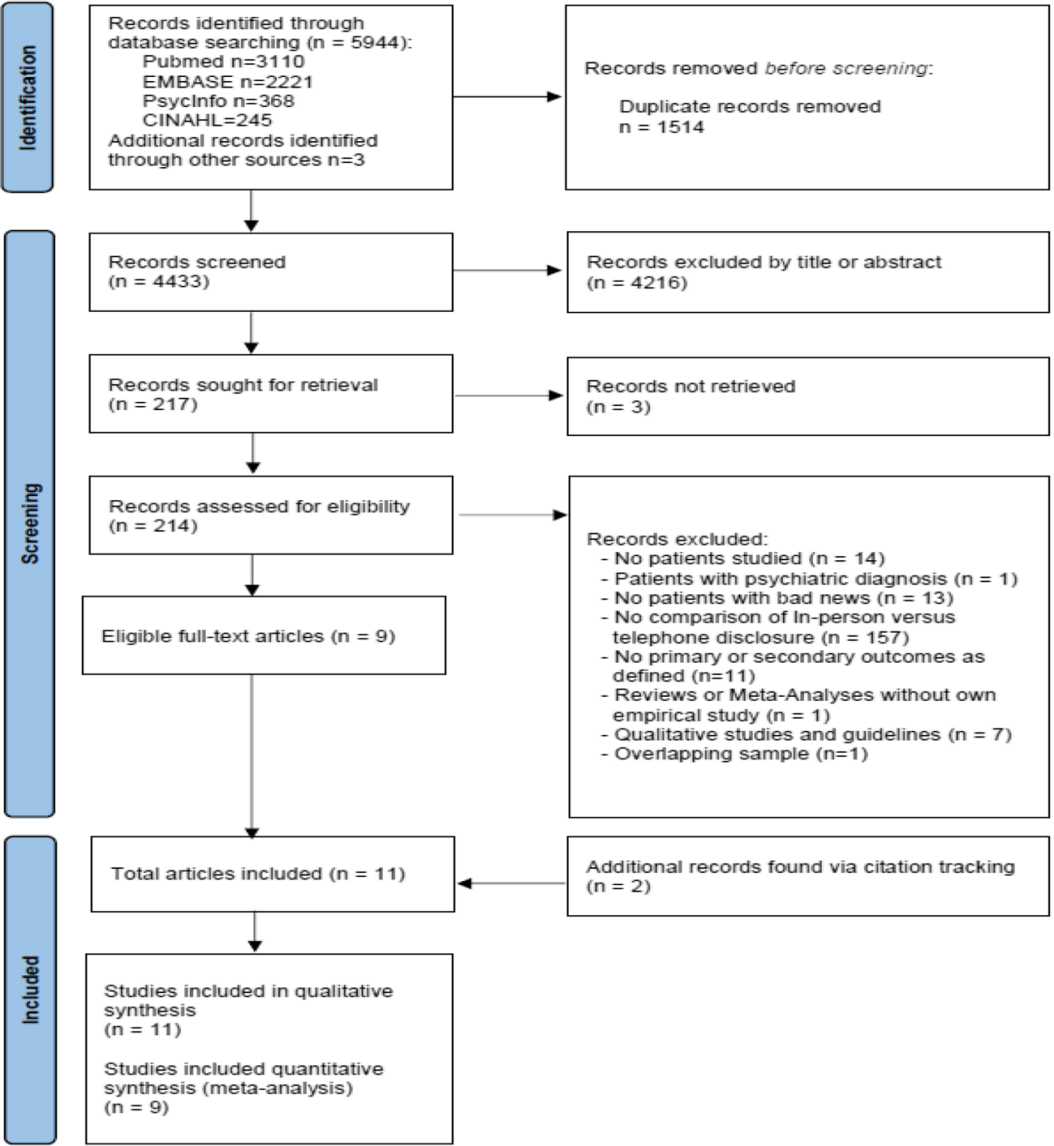
PRISMA flow diagram of the study selection process.

We identified 5944 records through the database search and three through citation tracking. After removing 1514 duplicates, we screened 4433 records based on titles and abstracts and in the process excluded 4216. Two reviewers independently reviewed the full texts of 214 articles and were thus able to include nine. There was one additional eligible article^17^ that we did not include as it reported a secondary analysis of a study we had already included,^18^ with the same analyses and outcome parameters, only at different follow-up time points. Citation tracking yielded two further records eligible for inclusion which led to 11 studies being included in the qualitative synthesis and 9 in the meta-analysis **(**Fig. 1**)**.

### Description of Studies

The 11 included studies were published between 1997 and 2021 and predominantly conducted in the USA^10, 18–22^ as well as in Australia^23^ and European countries, i.e., the United Kingdom,^24^ the Netherlands,^25^ Germany,^15^ and Denmark.^26^ Study sample sizes ranged from 24 to 434 participants. Seven studies evaluated the disclosure of malignancy diagnoses such as breast cancer,^15, 22, 24^ gynecologic cancer [19], thoracic cancer,^26^ melanoma,^23^ and different types of cancer.^10^ The remaining 4 studies assessed breaking bad news of genetic testing results for high-risk constellations of hereditary breast, gynecological and/or gastrointestinal cancer,^18, 21^ Alzheimer disease^20^ and hypertrophic cardiomyopathy.^25^

All studies investigating psychological distress used well-established and validated questionnaires. Assessment of satisfaction varied broadly from singular binary or categorical items to scales and qualitative interviews. Follow-up times ranged from several days to years.

The study characteristics and main findings are presented in Table 1.

**Table 1:** Summary of studies included in the systematic review

Authors	Study Purpose	Country	n participants analyzed	n in-person	n tele-phone	Participants	Design	Methods	Results
Bradbury et al., 2018	To understand the risks and benefits of telephone disclosure and to test noninferiority of telephone vs. in-person disclosure of genetic test results regarding patient-relevant outcomes	USA	88	41	47	Patients enrolled in clinical cancer genetic programs with any clinical genetic testing for hereditary breast, gynecological, and/or gastrointestinal cancer syndromes 1711 eligible, 208 (17.7%) declined due to strong preference for in-person disclosure Telephone: 473 randomized, 324 analyzed, 261 with follow-up data including 47 who received bad news In person: 497 randomized, 37 declined intervention, 284 analyzed, 168 with follow-up data including 41 who received bad news	Multicenter randomized noninferiority trial	All participants completed a baseline survey at the end of their in-person pretest counseling session and were sent a follow-up survey within 7 days after disclosure	**Anxiety symptoms:** Patients with a positive test result that was disclosed by telephone had a greater decrease in general anxiety (mean change score –0.37 [SD 2.26]) compared to those with in-person disclosure (mean change score + 0.87 [SD 2.64]) according to primary (p = 0.02) but not secondary analyses utilizing multiple imputations (p = 0.07). Change in state anxiety did not differ between patients with telephone (+ 1.81 [SD 7.58]) vs. in-person disclosure (+ 4.73 [SD 11.41]; p > 0.05) **Depressive symptoms:** Change in depressive symptom levels did not differ between patients with telephone (-0.23 [SD 1.81] vs. in-person disclosure (+ 0.24 [SD 3.00]; p > 0.05) **PTSD symptoms:** Change in PTSD symptom levels did not differ between patients with telephone (+ 1.88 [SD 9.73]) vs. in-person disclosure (+ 4.22 [SD 8.58]; p > 0.05) **Satisfaction:** Change in satisfaction levels did not differ between patients with telephone (-3.02 [SD 3.78]) vs. in-person disclosure (-3.16 [SD 4.96]; p > 0.05)
Christensen et al., 2018	To evaluate noninferiority of telephone vs. in-person disclosure of genetic test results regarding patient-relevant outcomes	USA	83	44	39	Individuals of the general population 25% of which had one relative affected with Alzheimer's Disease. Exclusion criteria: Severe anxiety or depression, histories suggestive of hereditary Alzheimer's disease, and cognitive impairment (modified Mini-Mental State Examination (education-adjusted) > 87). Telephone: 141 randomized, 125 analyzed, 39 had received bad news In-person: 149 randomized, 132 analyzed, 44 had received bad news	Multicenter randomized noninferiority trial	Participants completed a phone interview, written questionnaire and received educational material prior to the genetic testing. Outcomes were assessed at the 6-week, 6-month and 12-month follow-up appointments through self-administered questionnaires. Only at the end of the follow-up appointments, genetic counselors reminded participants about their genotypes and the associated risks. Those with concerning anxiety or depression scores were immediately interviewed by a genetic counselor	Telephone disclosure was not associated with increased symptoms of anxiety, depression or PTSD 6 weeks and 6 months after baseline compared to in-person disclosure, i.e. showed non-inferiority, within the subset of participants who received "bad news", i.e. learned that they were APOE ε4 carriers. Participants with telephone disclosure reported more symptoms of depression and PTSD but not anxiety at 12-month follow-up compared to those with in-person disclosure. However, mean anxiety and depression scores were remarkably below cut-off scores for clinical concern
Kinney et al., 2016	To examine if telephone genetic counseling is noninferior to in-person counseling regarding long-term outcome, i.e. psychosocial, informed decision-making, quality of life, and risk management outcomes at 1-year follow-up	USA	24	12	12	English-speaking women, 25 to 74 years of age, Utah residents with personal/family histories meeting hereditary breast and ovarian cancer genetic testing guidelines, with telephone access, who could travel to in-person counseling at one of 14 clinics, and had no prior genetic counseling and/or BRCA1/2 testing Telephone: 12 had received bad news In-person: 12 had received bad news	Singe-center Randomized noninferiority trial	Sub-analysis of a larger randomized noninferiority trial. Participants were randomized to either in-person or telephone counseling. Those who decided to have genetic testing received their result according to their randomization. Outcomes were assessed via telephone, internet or mailed surveys at baseline, 1 week after pre-test and post-test counseling, 6 months, and 1 year after the last counseling session. This study focuses on 1-year outcomes	**Anxiety symptoms:** There was no significant change in anxiety symptom levels from baseline to 1-year follow-up within patients with telephone disclosure (n = 12; mean change score 0.33 [95% CI -0.87 to 1.35]) and those with in-person disclosure (n = 12; mean change in score -0.25 [95% CI [-2 to 1.82]). Further, there was no difference in change scores between groups (mean difference 0.58 [95% CI -3.64 to 2.33]) **PTSD symptoms:** There was no significant change in PTSD symptom levels from baseline to 1-year follow-up within patients with telephone disclosure (n = 11; mean change score -2.09 [95% CI -9.01 to 5.54]) and those with in-person disclosure (n = 12; mean change score 2.25 [95% CI -8 to 1.79]). Further, there was no difference in change scores between the two groups (mean difference 0.16 [95% CI -15.61 to 7.71]) Due to the small numbers of BRCA1/2 positive participants, the interpretability of noninferiority of telephone counseling versus in-person counseling is limited
Bodtger et al., 2021	To investigate the effect of telephone vs. in-person disclosure of a cancer diagnosis on psychosocial consequences four weeks later	Denmark	170	87	83	Patients with suspicious lesions in lung, pleura or mediastinum with a pulmonologist's judgement of indication for invasive workup and expected survival of > 1 month. Of 492 eligible patients, 151 (31%) could not accept randomization Telephone: 129 randomized, 83 (64%) analyzed In person: 126 randomized, 87 (69%) analyzed 97 of 255 participants (38%) were female	Single-center randomized controlled trial	Patients underwent a cancer workup including several steps: 1. telephone call on symptoms, results, clinical suspicion and plan, 2. advanced imaging and telephone call on results, suspicion and plan, 3. invasive workup in bronchoscopy suite in person, randomization, baseline assessment, and 4. result disclosure in person or via telephone call Patients received the study questionnaires via mail at baseline (3rd step) and 4 weeks after receiving the final result	Patients with telephone disclosure did not have increased anxiety or depression at 4 week-follow-up compared to those with in-person disclosure Negative delta values equal higher symptom levels in the telephone group **Anxiety symptoms:** There was no significant difference between anxiety levels in patients with telephone vs. in-person disclosure (delta 1.03 [95% CI -0.67 to 2.74], p = 0.24) **Depressive symptoms:** There was no significant difference between depressive symptom levels patients with telephone vs. in-person disclosure (delta 0.56 [95% CI -1.03 to 2.16], p = 0.49) **Other:** No statistically significant intra-group differences in the following domains: behavior, dejection, negative impact on sleep/relaxation, social network, existential values, impulsivity, empathy, being regretful of still smoking
Schofield et al., 2003	To evaluate which recommended communication strategies for disclosing a new diagnosis of melanoma are associated with higher patient satisfaction and less psychological distress	Australia	131	42	89	Patients with melanoma diagnosed by biopsy within the preceding 4 weeks. Of 150 eligible patients 14 refused to participate, 3 had died; 131 with data on psychological distress. English speaking and contactable by telephone	Prospective cohort study	Consecutive new patients who visited the specialized Melanoma Unit for treatment or clinical opinion from a surgeon to whom a new melanoma diagnosis had been disclosed, completed psychometric questionnaires assessing symptoms of psychological distress, i.e. anxiety and depression, and a tailored questionnaire on satisfaction with their consultation at three follow-up time points, i.e. 4, 8 and 17 months after baseline. Secondary outcomes included communication experiences and preferences. Patients were contacted and asked for informed consent 1–2 months after the disclosure. Patients completed the questionnaires, which were sent by mail, at home	There was no difference in psychological distress and satisfaction between patients with telephone vs. in-person disclosure at any follow-up time point **Anxiety symptoms:** There was no significant difference in mean anxiety scores between patients with telephone vs. in-person disclosure 4 months (4.05 vs. 4.65, p > 0.05), 8 months (4.50 vs. 5.45, p > 0.05) and 17 months (4.58 vs. 5.31, p > 0.05) later **Depressive symptoms:** There was no significant difference in mean depressive symptom scores between patients with telephone vs. in-person disclosure 4 months (2.16 vs. 2.42, p > 0.05), 8 months (2.32 vs. 2.89, p > 0.05) and 17 months (2.39 vs. 2.85, p > 0.05) later **Satisfaction:** There was no significant difference in the proportions of patients who were satisfied (63% of patients with telephone vs. 64% of patients with in-person disclosure, p > 0.05)
Brake et al., 2007	To investigate how physicians communicate a new breast cancer diagnosis and the relative's role in this communication process. Specifically, to examine how information consistency and presence of relatives during disclosure relate to satisfaction. Further, to investigate the path German breast-cancer patients take until receiving their final diagnosis	Germany	222	187	35	Participants were women aged 70 years or younger with a first manifestation of breast cancer (stages T1-T3; N0-N2; no evidence of metastases) excluding women with multiple cancers, recurrences of breast cancer and any psychiatric diagnosis. Of 360 patients, 125 (35%) refused to participate and 222 (62%) with complete data were included	Prospective cohort study	The present study was part of an ongoing prospective study on the role of psychosocial factors in the course of breast cancer in three gynecological clinics. Patients were approached after surgery of breast cancer and asked to participate in the long-term study with five follow-up assessments. For this analysis, data was derived from the first assessment period where semi-structured, tape-recorded interviews with patients were conducted within 6 weeks after surgery and additional information was extracted from medical records	**Satisfaction:** Patients with telephone disclosure were not more likely to be dissatisfied than those with in-person disclosure (OR 2.5 [95% CI 0.8 to 7.5] p = 0.12) A higher odds ratio indicates a higher likelihood of dissatisfaction in patients to whom the bad news were conveyed by telephone
Figg et al., 2010	Investigation of how different cancer diagnoses were disclosed to patients and the impact thereof on patient satisfaction	USA	434	355	79	Participants were adult English-speaking patients who had previously received a diagnosis of cancer at different outside facilities in varying settings. Of 460 patients invited to participate in the study, 437 completed the study assessment and provided signed consent	Retrospective survey	The study included patients who had been referred to the National Cancer Institute and had already previously been told their diagnosis at another institution. Participants' experience of diagnosis disclosure was assessed through a self-administered questionnaire	**Satisfaction:** Telephone disclosure was associated with lower mean satisfaction scores compared to in-person disclosure ([mean 47.2, standard error of mean 3.7] vs. [mean 68.2, standard error of mean 1.6], p for group interaction < 0.001). Factors associated with higher satisfaction included personal setting as location of disclosure, length of disclosure > 10 min and discussion of treatment options **Trust in physician:** Telephone disclosure was not associated with lower or higher trust in physician compared to in-person disclosure (data not reported). Longer discussions and inclusion of treatment options were associated with higher trust
Cantril et al., 2019	To investigate patient experiences and preferences regarding the disclosure of a breast cancer diagnosis and to evaluate the role of the breast nurse navigator during the diagnostic experience	USA	177	93	84	English-speaking patients who had been diagnosed with breast cancer at one of four breast cancer centers. Of 517 patients who received the survey, 199 (38%) participated	Retrospective survey	The study was conducted in the context of a quality improvement survey that was sent to patients who had been diagnosed with breast cancer diagnosis at one of four breast cancer centers	**Satisfaction:** Telephone disclosure was associated with lower satisfaction compared to in-person disclosure (n = 25 [30%] vs. n = 12 [13%] patients with low satisfaction, *p* = 0.002) **Other:** Half of the patients with telephone disclosure did not have a preference regarding telephone or in-person disclosure and 28% would have preferred in-person disclosure. The majority (77%) of those with in-person disclosure preferred this method and only two found telephone disclosure the ideal method. Two important themes mentioned by many patients in the open-ended questions were "Just need to know/know as soon as possible." (n = 81, 32%) which was more likely to be mentioned by patients who preferred or were neutral regarding telephone disclosure (p < 0.001) and "A personal touch/emotional support." (n = 62, 24%), more likely to be mentioned by those who had a preference or were neutral regarding in-person disclosure (*p* < 0.001)
Kuroki et al., 2013	To investigate gynecologic oncology patients' experience of cancer diagnosis disclosure. Additionally, to evaluate patients' anxiety levels at diagnosis disclosure	USA	93	70	23	Convenience sample of 100 English-speaking adult patients diagnosed with a gynecologic cancer (cervical, endometrial, fallopian tube, ovarian, peritoneal, and vaginal or vulvar cancer) within the preceding 6 months. Patients with recurrent disease were excluded. Of 120 eligible patients, 11 (9%) declined and 21 (18%) did not return the study questionnaire	Retrospective survey	The study was conducted at a comprehensive cancer center. Patients filled out a self-administered 83-item study questionnaire, either at the time of an outpatient appointment in a private room or at home and returned by mail	**Satisfaction:** Telephone disclosure was associated with lower patient satisfaction ratings (mean 72; SD 36.6) compared to in-person disclosure (mean 91.3 [SD 16.5]; median 90 vs. 100, p = 0.02)
Campbell et al., 1997	To investigate patient satisfaction with the method of disclosure, i.e. by telephone vs. in person, of results of biopsy for breast cancer	UK	101	68	33	Patients who underwent breast biopsy due to impalpable breast lesions previously detected in a mammographic screening. Of 202 women, 171 (85%) completed the study assessment. Of 101 patients in whom the biopsy had revealed a malignant disease, 33 had received this diagnosis by telephone and 68 in person	Retrospective survey	Participants completed a self-administered study questionnaire which had been sent to them by mail	**Satisfaction:** Participants to whom the bad news, i.e. malignant biopsy result, was disclosed by telephone shortly after the biopsy were more likely to be satisfied than those who received the result in person at a later date (88% vs. 66%, χ^2^ with Yates’ correction = 4.29, *p* < 0.05) **Other:** Communication of malignant results took place within 7 days after biopsy in 84% of participants with telephone disclosure and in 41% of those with in-person disclosure
Christiaans et al., 2009	To evaluate the attitudes and experience of patients undergoing comprehensive genetic counseling and testing for cardiomyopathy risk. The authors aimed to analyze associations between sociodemographic and clinical characteristics and patients' attitude towards cardiogenetic care	Netherlands	63	19	44	Hypertrophic cardiomyopathy mutation carriers who were relatives of 95 hypertrophic cardiomyopathy patients. Participants had to be Dutch-speaking and at an age ≥ 16 years. Of 143 carriers, 123 (86%) participated. Of those, 44 (36%) had received the DNA results by telephone, 19 (15%) in person and the remaining in other ways such as per mail	Cross-sectional study	Participants underwent multidisciplinary genetic counseling testing. The results were subsequently disclosed by telephone, in person or per mail. Outcomes were assessed by a self-administered questionnaire that was sent per mail	**Satisfaction:** Disclosure by telephone and mail was associated with higher satisfaction compared to in-person disclosure at the outpatient clinic (mean 93 [SD 14] & mean 91 [SD 21] vs. mean 84 [SD 21], *p* = 0.032) Overall, 102 (95%) were satisfied with their way of receiving the result and 4 (4%) would have preferred the outpatient clinic Satisfaction with counseling was significantly higher in mutation carriers who were older (*p* = 0.003), had a partner (*p* = 0.017), and/or comorbidity (*p* = 0.028)

n, number of participants; SD, standard deviation; PTSD, post-traumatic stress disorder; CI, confidence interval; OR, odds ratio

### Description of Findings of the Included Studies

#### Association of Disclosure of Bad News via Telephone vs. in Person with Psychological Distress

### Anxiety Symptoms

Five out of all 11 studies published in 2003 and 2016 to 2021 reported findings on the association of disclosure of bad news via telephone vs. in person with symptoms of anxiety at follow-up.^18, 20, 21, 23, 26^ None of the studies showed increased anxiety in patients to whom bad news were disclosed via telephone compared to those with in-person disclosure.

Three randomized controlled trials investigated the disclosure of a positive genetic test result. In the study of Bradbury et al.,^18^ patients with telephone disclosure showed a greater decrease in general anxiety, but not state anxiety, i.e. a transient emotional state, from baseline (pre-disclosure) to one week post-disclosure compared to those with in-person disclosure when raw data rather than the imputed data set was analyzed. In the study of Christensen et al.,^20^ telephone disclosure was non-inferior to in-person disclosure regarding anxiety symptoms at 6-week, 6-month and 12-month follow-up. In the study of Kinney et al.,^21^ anxiety symptom levels one year after baseline did not differ between patients with telephone vs. in-person disclosure.

Two studies, a randomized controlled trial^26^ and a prospective cohort study^23^ evaluated the disclosure of malignancy diagnoses, i.e. lung, mediastinal & pleural cancers and melanoma, respectively. Both did not reveal any differences between patients with telephone and in-person disclosure regarding anxiety symptom levels 4 weeks and 4, 8 and 17 months after baseline, respectively.

### Depressive Symptoms

Four out of 11 studies published in 2003 and 2016 to 2021 reported findings on the association of disclosure of bad news via telephone vs. in person with depressive symptoms at follow-up.^18, 20, 23, 26^ None found an association between telephone or in-person disclosure and depressive symptoms.

Two randomized controlled studies investigated disclosure of a positive genetic test result. In the study of Christensen et al.,^20^ telephone disclosure was inferior to in-person disclosure regarding depressive symptom levels at 12-month follow-up. There was no difference in depressive symptom levels between the two groups at 6-week and 6-month follow-up. Additionally, the average depressive symptom score was still well below the cutoff for clinical concern. In the study of Bradbury et al.,^18^ there was no association between telephone or in-person disclosure and change in depressive symptom levels from baseline to one week post-disclosure.

Two studies, a randomized controlled trial^26^ and a prospective cohort study^23^ evaluated disclosure of malignancy diagnoses, i.e. lung, mediastinal and pleural cancers and melanoma, respectively. Both did not reveal any differences in depressive symptom levels at 4 weeks and 4, 8 and 17 months after baseline, respectively.

### PTSD Symptoms

Three studies published in 2003, 2018 and 2021 reported findings on the association of disclosure of bad news via telephone or in person and PTSD symptom levels at follow-up with one study revealing an association.^18, 20, 21^ All 3 studies are randomized controlled trials in the field of genetic testing. In the study of Christensen et al.,^20^ telephone disclosure was inferior to in-person disclosure regarding PTSD symptom levels at 12-month follow-up. There was no difference in symptom levels between the two groups at 6-week and 6-month follow-up. Bradbury et al.^18^ found that the change in PTSD symptom levels from baseline to one-week follow-up did not differ between patients with telephone vs. in-person disclosure. In the study of Kinney et al.,^21^ there was no change in PTSD symptom levels from baseline to follow-up within both groups. PTSD symptom levels 12 months after baseline did not differ between patients with telephone vs. in-person disclosure.

### Association of Disclosure of Bad News via Telephone vs. in Person with Patient Satisfaction

Eight studies published in 1997 to 2019 reported findings on the association of disclosure of bad news via telephone vs. in person and patient satisfaction at follow-up with inconclusive results.^10, 15, 18, 19, 22–25^ In 3 of these, telephone disclosure was associated with lower satisfaction and in 2 with higher satisfaction. Lastly, 3 studies did not show any association between telephone or in-person disclosure and satisfaction.

Two studies evaluated disclosure of a positive genetic test result. In the randomized controlled trial of Bradbury et al.,^18^ there was no difference in patient satisfaction one week after disclosure between patients who received results via telephone vs. in person. In the cross-sectional study of Christiaans et al.,^25^ disclosure by telephone or mail was associated with higher patient satisfaction at 3-year follow-up compared to in-person disclosure.

Six observational studies evaluated satisfaction with disclosure of a new cancer diagnosis via telephone vs. in person. Three studies found that patients who received the diagnosis via telephone were less satisfied with disclosure compared to those who were told in person.^10, 19, 22^ Two studies did not reveal an association between telephone or in-person disclosure and patient satisfaction within the subsequent 6 weeks as well as 4, 8 and 17 months, respectively.^15, 23^ In the study of Campbell et al.,^24^ patients who received the bad news via telephone were more likely to be satisfied to have been informed this way than patients who were told in person.

### Trust in the Health Care Worker Disclosing the Bad News

The study of Figg et al.^10^ evaluated the association of disclosure of bad news via telephone vs. in person and patients’ trust in physician after result disclosure. Almost 80% of patients in this sample reported a greater than neutral level of trust and 16% said they had absolute trust. There was no association between level of trust and disclosure of bad news via telephone vs. in person.

### Quantitative Analysis

Nine studies with 1284 patients that evaluated breaking bad news via telephone compared to in person were included in the meta-analysis. Three studies reported results on psychological distress, i.e., anxiety, depression or PTSD, and 7 on satisfaction.

#### Anxiety Symptoms

Three studies (published between 2018 and 2021) including 285 patients evaluated symptoms of anxiety, 2 with a high risk of recruitment bias^18, 26^ and one with a low risk of bias.^20^ There was no mean difference regarding anxiety symptom levels when bad news was disclosed by telephone compared to in person (standardized mean difference [SMD] 0.10 [95% CI -0.15 to 0.35]) **(**Table 2**)**. There was little heterogeneity among trials (*I*^*2*^ = 13%, *p* = 0.32).

**Table 2:** Association between disclosure of bad news via telephone vs. in person and symptoms of anxiety

	Telephone		In person		Std. mean difference^1^	
Study	n	Mean (SD)	n	Mean (SD)	Weight	IV (95% CI)
Christensen et al. (2018)	39	3.9 (5.31)	44	2.55 (3.02)	29.5%	0.31 (-0.12, 0.75)
Bradbury et al. (2018)	47	35.46 (11.62)	41	37.36 (14.36)	31.3%	-0.15 (-0.56, 0.27)
Bodtger et al. (2021)	54	12.94 (5.18)	60	12.27 (4.46)	39.2%	0.14 (-0.23, 0.51)
Total	140		145		100%	**0.10 (-0.15, 0.35)**

^1^Analysis: Random-effects model calculating the standardized mean difference

Std. mean difference = standardized mean difference; n = number of patients in group; SD = standard deviation; IV = inverse variance; 95% CI = 95% confidence interval

#### Depressive Symptoms

Three studies (published between 2018 and 2021) including 284 patients evaluated depressive symptoms, 2 with a high risk of recruitment bias^18, 26^ and one with a low risk of bias.^20^ There was no mean difference in depressive symptom levels when bad news was disclosed by telephone compared to in person (SMD 0.10 [95% CI -0.30 to 0.49]) **(**Table 3**)**. There was substantial heterogeneity among trials (*I*^*2*^ = 64%, *p* = 0.06).

**Table 3:** Association between disclosure of bad news via telephone vs. in person and depressive symptoms

		Telephone		In person		Std. mean difference^1^
Study	n	Mean (SD)	n	Mean (SD)	Weight	IV (95% CI)
Christensen et al. (2018)	39	6.62 (8.16)	44	4.23 (4.56)	31.8%	0.36 (-0.07, 0.80)
Bradbury et al. (2018)	47	1.9 (2.44)	41	2.78 (3.18)	32.6%	-0.31 (-0.73, 0.11)
Bodtger et al. (2021)	53	12.13 (4.23)	60	11.20 (3.94)	35.6%	0.23 (-0.14, 0.60)
Total	139		145		100%	**0.10 (-0.30, 0.49)**

^1^Analysis: Random-effects model calculating the standardized mean difference

Std. mean difference = standardized mean difference; n = number of patients in group; SD = standard deviation; IV = inverse variance; 95% CI = 95% confidence interval

#### PTSD Symptoms

Two studies assessed symptoms of PTSD in 171 patients with a high risk of recruitment bias^18^ and low risk of bias,^20^ respectively. There was no mean difference in symptom levels of PTSD when bad news was disclosed by telephone compared to in person (SMD -0.01 [95% CI -0.48 to 0.36]) **(**Table 4**)**. Heterogeneity between trials was low (*I*^*2*^ = 0%, *p* = 0.74).

**Table 4:** Association between disclosure of bad news via telephone vs. in person and symptoms of post-traumatic stress disorder

	Telephone		In person		Std. mean difference^1^	
Study	n	Mean (SD)	n	Mean (SD)	Weight	IV (95% CI)
Christensen et al. (2018)	39	6.41 (9.66)	44	5.98 (9.47)	48.6%	0.04 (-0.39, 0.48)
Bradbury et al. (2018)	47	18.03 (13.02)	41	18.80 (13.73)	51.4%	-0.06 (-0.48, 0.36)
Total	86		85		100%	**-0.01 (-0.48, 0.36)**

#### Satisfaction

**Figure 2:**
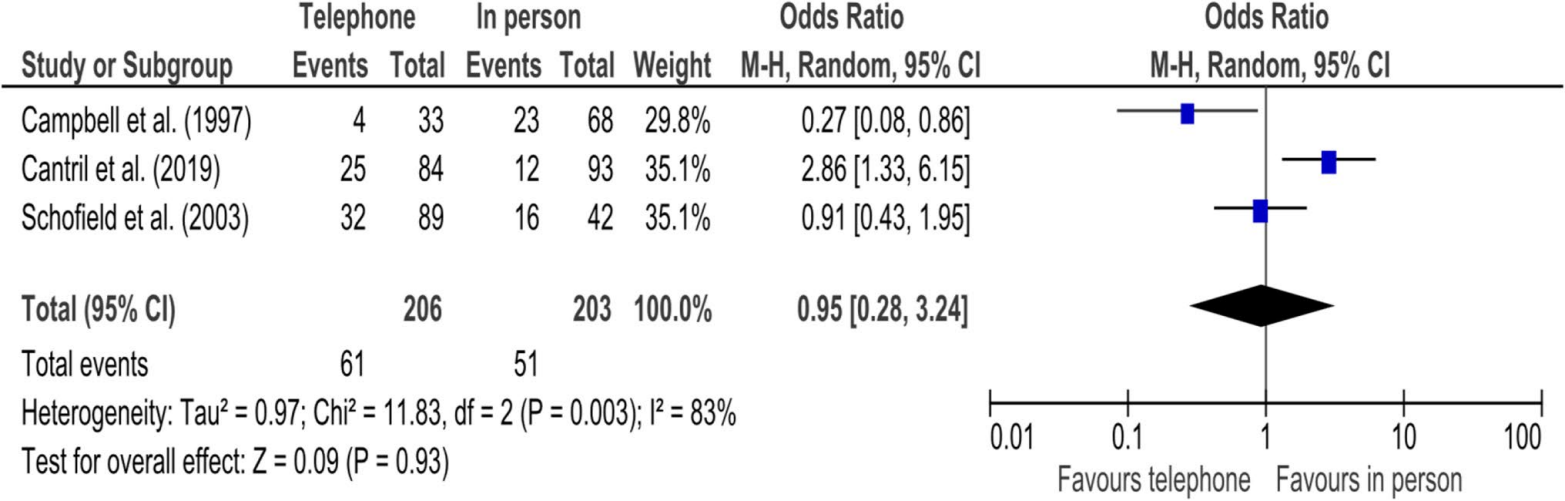
Forest plot showing the association between disclosure of bad news via telephone vs. in person and patient satisfaction. Legend: The squares and horizontal lines correspond to the study-specific odds ratios (OR) and 95% confidence intervals (CI), respectively. The diamond represents the pooled OR of satisfaction. Abbreviations: M-H = Mantel–Haenszel method.

Seven studies (published between 2009 and 2019) with mostly high risk of bias evaluated satisfaction with four studies including 678 patients assessing satisfaction levels^10, 18, 19, 25^ and 3 studies with 409 participants comparing the proportions of patients who were satisfied with the way bad news were disclosed.^22–24^ There was no mean difference in satisfaction levels when bad news were disclosed by telephone compared to in person (SMD -0.29 [95% CI -0.83 to 0.25]) **(**Table 5**)**. Further, risk for low satisfaction in patients who received bad news by telephone was similar compared to those with in-person disclosure (OR 1.00 [95% CI 0.26 to 3.84]) **(**Fig. 2**)**. Heterogeneity among these trials was high (*I*^2^ = 87%, *p* = 0.0005).

**Table 5:** Association between the disclosure of bad news via telephone vs. in person and satisfaction levels

	Telephone		In person		Std. mean difference^1^	
Study	n	Mean (SD)	n	Mean (SD)	Weight	IV (95% CI)
Christiaans et al. (2009)	44	93 (14)	19	84 (21)	22.8%	0.54 (0, 1.09)
Kuroki et al. (2013)	23	72 (36.6)	70	91.3 (16.5)	24.0%	-0.83 (-1.32, -0.34)
Bradbury et al. (2018)	47	35.64 (4.63)	41	36.05 (4.92)	25.2%	-0.09 (-0.50, 0.33)
Figg et al. (2010)	79	47.20 (32.89)	355	68.20 (30.15)	28.0%	-0.68 (-0.93, -0.44)
Total	193		485		100%	**-0.29 (-0.83, 0.25)**

Std. mean difference = standardized mean difference; n = number of patients in group; SD = standard deviation; IV = inverse variance; 95% CI = 95% confidence interval

^1^Analysis: Random-effects model calculating the standardized mean difference

## Discussion

There is a growing demand for telemedicine including the disclosure of bad news despite little insight regarding potential adverse effects. Therefore, in this systematic review and meta-analysis, we investigated if disclosure of bad news via telephone is associated with increased psychological distress and lower patient satisfaction compared to in-person settings. We included 11 studies in the qualitative synthesis and 9 in the meta-analysis. Our findings suggest that breaking bad news via telephone is neither associated with increased psychological distress nor lower patient satisfaction compared to breaking bad news in person.

Five studies^18, 20, 21, 23, 26^ evaluated the association between the disclosure of bad news via telephone compared to in-person disclosure and psychological distress. None of the studies revealed any significant association between mode of disclosure and psychological distress, i.e., symptoms of anxiety, depression or PTSD. These studies evaluated the disclosure of a new cancer diagnoses and genetic test result indicating a high risk for Alzheimer disease or a hereditary cancer syndrome. Although, the types of bad news might differ regarding their immediate impact on patient’s life, results were similar.

The uniformity of results within and across the studies assessing different contents of bad news and manifestations of psychological distress at different time points, suggests that breaking bad news via telephone is not associated with increased psychological distress and could be an acceptable alternative to in-person disclosure, at least in certain settings. Indeed, when in-person disclosure of bad news within a reasonable time period is not possible, e.g. if a patient lives far away or there is no available appointment in the near future, and at the same time, receipt of the bad news is either urgent or a delay could trouble patients due to uncertainty, telephone disclosure may be preferable over in-person disclosure.

So far, there are few expert recommendations on breaking bad news via telephone to patients^27–29^ but these are mostly based on clinical experience and studies on how to communicate bad news in general and there are no specific evidence-based recommendations.^5^ So far, the findings of the existing studies suggest that the modality of disclosure might play a secondary role and the way in which the bad news are communicated might be more important. This might include preparing patients for the possibility of receiving bad news beforehand and, at the time of the conversation, first ensuring that they are in an appropriate setting.^13, 28^ Further, the structure and content of the breaking bad news conversation may be relevant. Therefore, several communication strategies were developed.^6^ In all of the five studies evaluating psychological distress that we included, the bad news were disclosed by specifically trained staff, i.e. genetic counselors and physicians who had completed courses in patient communication during their specialist training.

Due to restrictions during the coronavirus pandemic, doctor-patient consultations via video-chat have become more common. In comparison to the disclosure of bad news over the phone, videoconferencing offers the opportunity for doctors to identify non-verbal communication and through this facilitate the recognition of patients’ emotional concerns.

Recently, proposed adaptations of existing breaking bad news communication strategies for telephone and videoconference disclosure, which were based on clinical experience and experimental pilot studies, have been published.^5, 13, 28–30^ These adaptations include recommendations on ensuring that patients are in an appropriate setting which may involve their significant others, exploring and acknowledging emotions verbally and expressing empathy through tone of voice. These need to be complemented by further research on the topic to facilitate the development of evidence-based communication strategies and should also include virtual patient encounters.

While satisfaction among individual trials showed both, positive and negative associations with bad news disclosed over the phone compared to in-person disclosure, in the quantitative analysis there was no statistically significant difference. This may be explained by patient preferences regarding mode of disclosure. Yet, most of the included studies did not report patients’ preferences. Of note, two studies evaluating disclosure of genetic test results ^18, 26^ reported that a significant number of patients declined participation due to a preference for one of the two disclosure modes. In the study of Bradbury et al., ^18^ conducted in the United States, almost 20% of patients declined participation due to a preference for in-person disclosure. The study of Bodtger et al.^26^ which was conducted in Denmark, reported that 151 (31%) patients did not agree to randomization and 105 (70%) of those chose to receive their genetic test result via telephone. Patients’ preference regarding the mode of disclosure and the involvement in decision-making^31^ might be associated with their satisfaction with the disclosure and should be evaluated in further research. Further, several studies revealed that other factors such as length of the conversation and discussion of treatment options were associated with patient satisfaction.^10, 19^ One study assessed patients’ level of trust.^10^ This study reports that almost 80% of patients had greater than neutral trust in their clinician and longer conversations and the discussion of treatment options were associated with high trust. Patients’ trust in their physician following a disclosure of bad news might be less depent on the mode of disclosure but rather on the quality of the relationship and the way in which the bad news are communicated, e.g. with the physician showing empathy and offering support. This might further emphasize the importance of the quality of the breaking bad news conversation irrespective from mode of disclosure.

There is a wide range of publication years across the studies included for the evaluation of the secondary endpoint, i.e. patient satisfaction. Importantly, telemedicine has recently become more popular and patients may thus be more used to telephone consultations today compared to some time ago. This may impact the generalizability of results to today’s standard of care. The wide range of publication years across the studies on the association between mode of disclosure and patient satisfaction as well as the changing role of telephone consultations is an important point that needs to be considered. As telephone consultations even for difficult conversations have become much more common in many countries since, patients might perceive this as usual care, potentially impacting patient satisfaction.

### Limitations

The 11 studies we were able to include, were heterogeneous regarding study design, patient populations, content of bad news, e.g. cancer diagnosis and increased genetic risk for a certain disease, and follow-up durations. This was especially true for the studies on our secondary outcome, i.e. satisfaction, which were also published over a time span of over 20 years. Due to this heterogeneity, generalization of our findings on the association between mode of disclosure and patient satisfaction is limited and further research is needed to confirm our findings. However, regarding psychological distress, 4 out of 5 studies included in the meta-analyses were RCTs with a methodologically sound study design published very recently between 2018 and 2021.

Three studies evaluated the disclosure of a high genetic risk for a certain disease or illness and two studies assessed the disclosure of a new malignancy diagnosis. Bad news are considered information that can potentially influence a patient’s life in some negative or unfavorable way. Still, the impact on patients’ psychological distress and satisfaction might differ. Due to the small number of identified studies, we were not able to analyze these two groups of studies separately.

Further, the use of telephone consultations including the disclosure of bad news has changed significantly since the beginning of the COVID-19 pandemic. While it was an option for patients who lived in great distance or preferred to be informed via telephone, during the COVID-19 pandemic suddenly it was often the only possible option. As all studies included in our systematic review were conducted before the beginning of the COVID-19 pandemic, this might limit the transferability of our findings to current times.

None of the studies calculated multivariable models including relevant covariates. As telephone disclosure of bad news is more common in the USA due to often great geographical distance between patient and treating healthcare worker, it might less likely lead to increased psychological distress and lower satisfaction. Our findings did not support this hypothesis. However, due to the small number of included studies, it is not possible to draw further conclusions.

### Strengths

Based on an extensive literature search, this systematic review and meta-analysis presents the current state of research on relevant patient-related outcomes associated with breaking bad news via telephone compared to in person.

The disclosure of bad news is a relevant part of clinical practice and one of the most publicized topics in the field of communication in healthcare. According to Pubmed, more than 2000 articles regarding the disclosure of bad news have been published since the year 2000. However, empirical studies are still scarce and recommendations on the communication of bad news are mainly based on expert opinions and clinical experience. Interestingly, we only found 11 studies and only 4 RCTs investigating the effect of face-to-face compared to over the phone disclosure of bad news.

It is worth mentioning that we did not find any study on the disclosure of bad news to adult patients’ relatives in our systematic review. Research has shown that the way healthcare professionals communicate with relatives of patients that are dying may influence their long-term psychological well-being.^32^ Breaking bad news to relatives frequently occurs in case of severe medical conditions such as an accident, acute deterioration or death of the patient. In these situations, the disclosure of the bad news is usually more time-sensitive and more likely to be conducted over the phone. Hence, rigorous studies on how to disclose bad news with patients and relatives in person, over the phone or virtually are warranted.

### Summary and Conclusions

Our findings suggest that disclosure of bad news via telephone compared to in-person disclosure does not lead to increased short- or long-term psychological distress, i.e. symptoms of anxiety, depression and PTSD or to lower satisfaction.

Since the beginning of the Covid-19 pandemic, there was an important increase of telephone disclosure of bad news as it was often the only available option when in-person consultations were not possible due to restrictions related to the risk of infection. Our results suggest that disclosure of bad news via telephone might be acceptable for patients and might not have adverse effects if the disclosure is well-conducted. Further insight on the association between the disclosure of bad news via telephone vs. in person as well as the role of other factors is needed to facilitate evidence-based recommendations and guidance for these challenging conversations.

### Supplementary Information

Below is the link to the electronic supplementary material.

Supplementary file1 (DOC 350 KB)

## Data Availability

Data are available upon request.

## Declarations

### Registration and Protocol

We pre-registered our review in the International Prospective Register of Systematic Reviews (PROSPERO; ID: PROSPERO 2021 CRD42021233266). The review protocol is available from the authors upon request.
