# Supplementary material for: The Disclosure of Bad News Over the Phone vs. in Person and its Association with Psychological Distress: a Systematic Review and Meta-Analysis
Source: J Gen Intern Med. 2023 Aug 8;38(16):3589–603. doi: 10.1007/s11606-023-08323-z (PMC10713955; doi:10.1007/s11606-023-08323-z)
Supplement: Supplementary file 1 — Supplementary file1 (DOC 350 KB) [file 11606_2023_8323_MOESM1_ESM.doc]

**Supplemental Online Content**

**Supplementary methods**

**Development of the search strategy**

**Search strategy**

**Risk of bias evaluation**

**MOOSE checklist**

**Supplementary results**

**eFigure 1.** Risk of bias assessment of the included studies

**eTable 1.** Summary of interventions, outcomes and measures of the included studies

**Supplementary methods**

**Development of the search strategy**

Initial search terms were drawn from a small sample of key articles. We used an iterative process to build the search strategy by running the search, scanning the relevant articles for additional terms, and then rebuilding the search strategy with the newly established relevant terms and related Medical Subject Heading (MeSH) terms.

**Search strategy** (Embase)

('interpersonal communication'/mj OR disclos*:ti,ab OR inform:ti,ab OR informed:ti,ab OR tell:ti,ab OR told:ti,ab OR communicat*:ti,ab) AND ('bad news':ti,ab OR 'genetic counseling'/mj OR 'genetic counseling':ti,ab OR 'death'/mj OR death:ti,ab OR 'dead'/mj OR dead:ti,ab OR die:ti,ab OR died:ti,ab OR decease*:ti,ab OR 'fatal outcome':ti,ab OR 'end of life'/mj OR 'end of life':ti,ab OR 'homicide'/mj OR 'life event'/mj OR 'diagnosis'/mj OR diagnosis:ti,ab OR diagnosing:ti,ab) AND ('telephone'/mj OR telephone:ti,ab OR phone:ti,ab OR 'cell phone use'/mj) AND ([adult]/lim OR [young adult]/lim OR [middle aged]/lim OR [aged]/lim OR [very elderly]/lim)

**Risk of bias evaluation**

In studies that were no randomized controlled trials, we only assessed the domains attrition bias (incomplete outcome data), reporting bias (selective outcome reporting) and other sources of bias. If risk of bias was judged as high in at least one of these domains, the study was considered at high risk of bias regarding the respective outcome. If all domains were rated low risk, the outcome was considered at low risk of bias. If information was insufficient to assess risk of bias, the study outcome was considered to be at unclear risk of bias.

**MOOSE Checklist for Meta-analyses of Observational Studies**

| **Item No** | **Recommendation** | **Reported on Page No** |
| --- | --- | --- |
| Reporting of background should include | | |
| 1 | Problem definition | 4 |
| 2 | Hypothesis statement | n.a. (explorative) |
| 3 | Description of study outcome(s) | - |
| 4 | Type of exposure or intervention used | 4 |
| 5 | Type of study designs used | 5 |
| 6 | Study population | 4, 5 |
| Reporting of search strategy should include | | |
| 7 | Qualifications of searchers (eg, librarians and investigators) | 6 |
| 8 | Search strategy, including time period included in the synthesis and key words | 5, Suppl. |
| 9 | Effort to include all available studies, including contact with authors | 6 |
| 10 | Databases and registries searched | 5 |
| 11 | Search software used, name and version, including special features used (eg, explosion) | n.a. |
| 12 | Use of hand searching (eg, reference lists of obtained articles) | 5 |
| 13 | List of citations located and those excluded, including justification | Fig. 1 |
| 14 | Method of addressing articles published in languages other than English | n.a. |
| 15 | Method of handling abstracts and unpublished studies | 5 |
| 16 | Description of any contact with authors | n.a. |
| Reporting of methods should include | | |
| 17 | Description of relevance or appropriateness of studies assembled for assessing the hypothesis to be tested | 7 |
| 18 | Rationale for the selection and coding of data (eg, sound clinical principles or convenience) | n.a. |
| 19 | Documentation of how data were classified and coded (eg, multiple raters, blinding and interrater reliability) | 6 |
| 20 | Assessment of confounding (eg, comparability of cases and controls in studies where appropriate) | eFigure 1 |
| 21 | Assessment of study quality, including blinding of quality assessors, stratification or regression on possible predictors of study results | eFigure 1 |
| 22 | Assessment of heterogeneity | 10, 11 |
| 23 | Description of statistical methods (eg, complete description of fixed or random effects models, justification of whether the chosen models account for predictors of study results, dose-response models, or cumulative meta-analysis) in sufficient detail to be replicated | 6 |
| 24 | Provision of appropriate tables and graphics | Figure 1; Table 1-4; eFigures 1&2, eTables 1&2 |
| Reporting of results should include | | |
| 25 | Graphic summarizing individual study estimates and overall estimate | eTable 2 |
| 26 | Table giving descriptive information for each study included | Table 1 |
| 27 | Results of sensitivity testing (eg, subgroup analysis) | n.a. |
| 28 | Indication of statistical uncertainty of findings | 10, 11 |

*From*: Stroup DF, Berlin JA, Morton SC, et al, for the Meta-analysis Of Observational Studies in Epidemiology (MOOSE) Group. Meta-analysis of Observational Studies in Epidemiology. A Proposal for Reporting. *JAMA*. 2000;283(15):2008-2012. doi: 10.1001/jama.283.15.2008.

| **Item No** | **Recommendation** | **Reported on Page No** |
| --- | --- | --- |
| Reporting of discussion should include | | |
| 29 | Quantitative assessment of bias (eg, publication bias) | - |
| 30 | Justification for exclusion (eg, exclusion of non-English language citations) | n.a. |
| 31 | Assessment of quality of included studies | 14 |
| Reporting of conclusions should include | | |
| 32 | Consideration of alternative explanations for observed results | - |
| 33 | Generalization of the conclusions (ie, appropriate for the data presented and within the domain of the literature review) | 12-15 |
| 34 | Guidelines for future research | 13, 15 |
| 35 | Disclosure of funding source | 29 |

**eFigure 1.** Risk of bias assessment of the included studies


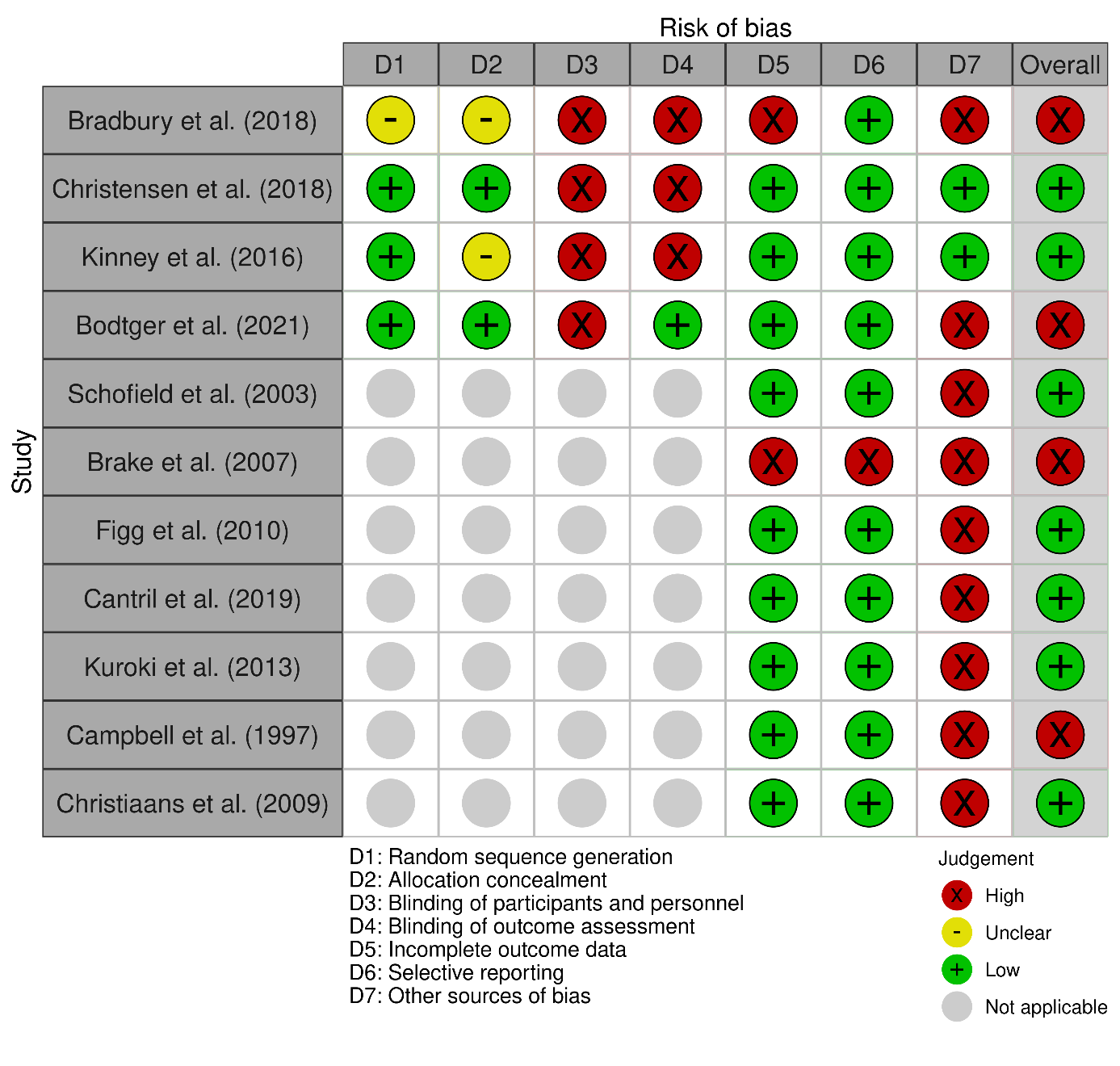


**eTable 1.** Summary of interventions, outcomes and measures of the included studies

| **Authors** | **Detailed communication / intervention elements** | **Outcomes and Measures** |
| --- | --- | --- |
| Bradbury et al., 2018 | Bad news were defined as positive genetic test result, indicating increased risk for specific cancer syndromes. Genetic test disclosures were delivered using standardized communication protocols, visual aids and checklists by 22 protocol-trained board-certified genetic or nurse counselors. Telephone disclosure sessions were scheduled and participants were recommended to return for in-person visit to further discuss medical management. In in-person disclosure sessions, medical management was discussed during the same visit. Participants received the follow-up survey via mail to complete within 7 days after disclosure. | **Anxiety symptoms:** General anxiety (preceding 7 days): Hospital Anxiety and Depression Scale (HADS), anxiety subscale; State anxiety (past 24 hours): State Trait Anxiety Inventory (STAI), state anxiety subscale.  **Depressive symptoms:** HADS, depression subscale  **PTSD symptoms:** Cancer-specific distress: 14 items of Impact of Events Scale (IES).  **Satisfaction:** measured with a nine-item scale evaluating participants' cognitive and affective perceptions of their genetic counseling and testing experience.  Analysis: Comparison of average change in symptoms and satisfaction from baseline (pre-test) to follow-up (within 7 days after disclosure) of patients with telephone vs. in-person disclosure (interaction change x group). |
| Christensen et al., 2018 | Bad news were defined as positive genetic test result, i.e. presence of the ε4 allele of APOE, indicating increased risk for Alzheimer's disease. Results were disclosed by genetic counselors according to randomization and using a script that also included information on Alzheimer's disease risk and allowed for questions or concerns from participants. | **Anxiety symptoms:** Beck Anxiety Inventory (BAI).  **Depressive symptoms:** Center for Epidemiological Studies–Depression Scale (CES-D).  **PTSD symptom**s: Impact of Events Scale (IES).  **Analysis:** Comparison of symptom levels at 6 weeks post-disclosure between patients with telephone vs. in-person disclosure. Non-inferiority was defined as a 99% CI margin of 5 points or lower. |
| Kinney et al., 2016 | In both groups, the pre-test counseling followed a standardized protocol using visual decision aids which were mailed in advance to those with telephone counseling and provided as a copy to the in-person group. Additionally, all participants received an educational brochure. Women in the in-person group who decided to have genetic testing could provide a sample at their appointment or bring a BRCA1/2 buccal test kit home while those with telephone counseling were mailed a test kit. Post-test counseling was performed by the same genetic counselor using tailored visual aids based on the results of the individual. Participants in both arms were mailed a letter summarizing their personalized risk assessment based on family history and/or genetic test result and management recommendations. | **Anxiety symptoms:** 6-item anxiety subscale of the Brief Symptom Inventory-18.  **PTSD symptoms:** 15-Item Impact of Event scale (IES).  **Analysis:** Comparison of average change in symptoms and satisfaction from baseline (pre-test) to 1-year follow-up of patients with telephone vs. in-person disclosure (interaction change x group). |
| Bodtger et al., 2021 | At the in-person appointment prior to the disclosure session, all patients were informed about previous and pending results, disclosure method, expected content of conversation, and that relatives/key persons are welcome to attend. The in-person group were provided with a written out-patient clinic appointment five working days later. Patients in the telephone group were informed that they will receive a telephone call between 08 AM and 5 PM on workdays, as soon as the results of invasive workup are available which is expectedly after 3 to 5 days. At the telephone call, patients are free to ask for a later call, to summon relatives, to turn on the loud speaker, and/or ask the physician to call one relative of choice to share the information.  **Disclosure:** At the session there was disclosure of diagnosis, what stage of cancer, referral for therapeutic intervention, if needed further diagnostic interventions. Whenever possible, the result was disclosed by a previously seen physician. | **Anxiety symptoms:** Anxiety subscale of the Consequences of Screening - Lung cancer (COS-LC) questionnaire. Measured as difference from baseline to 4-week follow-up.  **Depressive symptoms:** Dejection subscale of the COS-LC questionnaire. Measured as difference from baseline to 4-week follow-up. Other: Other domains of the COS-LC questionnaire.  **Analysis:** Linear regression models calculating between-groups difference of symptom levels at 4 weeks post-disclosure (telephone vs. in-person disclosure). |
| Schofield et al., 2003 | Almost all patients had their diagnosis disclosed to them either by their general practitioner, surgeon or other specialist physician. Information about the extent of the disease and treatment options were also communicated. In 39% of cases the family of the patient was present. Most participants with in-person disclosure were told in a private room. 37% of participants were able to discuss follow-up questions wihtin a day and 80% within a week. | **Anxiety symptoms:** Hospital Anxiety and Depression Scale - Anxiety subscale (HADS-A).  **Depressive symptoms:** Hospital Anxiety and Depression Scale - Depression subscale. Satisfaction: Self-developed questionnaire assessing satisfaction with counseling. The results were dichotomized in high-mid vs. low satisfaction. Other: comments about communication experiences (at 4 months only).  **Analysis:** Comparison of mean anxiety and depression scores between patients with telephone vs. in-person disclosure at 4-, 8- and 17-month follow-up, respectively. Comparison of the proportions of satisfied patients at 4 months. |
| Brake et al., 2007 | The physician disclosed the diagnosis on the phone or in person. | **Satisfaction:** Patient satisfaction was determined according to statements made during tape-recorded interviews and coded into dissatisfied, in-between and satisfied.  **Analysis:** For logistic regression analysis, the Odds Ratios for being dissatisfied were computed against the other categories taken together. Age was included as a covariate. |
| Figg et al., 2010 | Before referral to the National Cancer Insitute and inclusion in the study, participants had been diagnosed with cancer in any setting in any US American country (or outside of the US in 8 patients). In 79 (18%) of included patients the diagnosis was disclosed by telephone and in 355 (82%) in person. Of the latter, 233 (66%) were told in the physician's office and 122 (34%) in the hospital. In similar proportions of patients, the cancer diagnosis was dislosed by a primary care physician, oncologist, surgeon or other physician. A minority received their diagnosis by a non-physician. In 72% of cases the spouse of the participant was present during the disclosure. Most sessions lasted 1-10 (36%) and 11-30 minutes (35%). | **Satisfaction**: Patients rated their satisfaction on a scale from 0 to 100.  **Trust in physician:** Wake Forest Trust Scale (short-form); sum score ranges from 5 to 25 points. Scores between 5 and 15 constitute lower and scores 16 to 25 higher trust.  **Analysis:** Mean satisfaction scores as well as number of patients with lower trust were compared among patients with telephone vs. in-person disclosure. |
| Cantril et al., 2019 | In 3 of 4 centres a breast nurse navigator was involved in the care of the patient including the disclosure of the diagnosis. In the in-person disclosure group, this breast nurse navigator disclosed the bad news. In the telephone group, the diagnosis was usually disclosed by the radiologist. Other professionals who disclosed the diagnosis included radiologists, surgeons, primary care providers, obstetricians/gynecologists and breast nurse navigators (in the telephone group). | **Satisfaction:** One survey item assessing how helpful patients found the method of disclosure (by telephone vs. in-person) with the response categories "not helpful at all", "neutral", "somewhat helpful" and "extremely helpful".  **Analysis:** The satisfaction item was dichotomized in "somewhat or extremely helpful" indicating higher satisfaction and "neutral or not at all helpful" indicating lower satisfaction. |
| Kuroki et al., 2013 | The settings in which the cancer diagnosis had been disclosed varied among participants. In most cases, a gynecologist or gynecologic oncologist disclosed the diagnosis in their office. Patients reported that these conversations lasted between 1-10 minutes (48%) and 11-30 minutes (46%). Gynecologic oncologists almost always disclosed the diagnosis in person (96%), among other physicians this rate was 63 to 75%. A disproportionately high amount (78%) of all telephone disclosures were performed by gynecologists. | **Satisfaction:** Patients were asked to retrospectively rate their satisfaction regarding the overall experience at the time of diagnosis disclosure on a scale from 1-100.  **Analysis:** Comparison of mean satisfaction ratings of patients with telephone vs. in-person disclosure (Wilcoxon rank-sum test) |
| Campbell et al., 1997 | All participants had an in-person appointment before the biopsy and had been prepared for all possible outcomes. Biopsy results were either disclosed by telephone shortly after the biopsy or communicated in person at a later date. | Satisfaction: Patients were asked to retrospectively rate if they were satisfied or not satisfied with the way in which they heard their result. |
| Christiaans et al., 2009 | Disclosure of DNA test results took place in-person, by telephone or by mail. Participants chose the disclosure method based on their preference except in case of psychosocial problems or additional questions arising at the pre-test appointment where it was preferred by counselors to communicate the result in person, often combined with a session with a social worker or psychologist. When the result was communicated by phone or mail, an additional appointment at the outpatient clinic was always offered. Psychosocial support was actively offered before and after DNA testing and also sessions with a social worker were available. | **Satisfaction:** A self-developed questionnaire evaluating attitudes towards genetic counseling and DNA testing across four domains, one of which was satisfaction with counseling. Scores for the various subscales were summed and rescaled to 0–100.  **Analysis:** Satisfaction of patients with in-person disclosure was compared with those with telephone or mail disclosure. |

**References**

1. McGuinness LA, Higgins JPT. Risk-of-bias VISualization (robvis): An R package and Shiny web app for visualizing risk-of-bias assessments. Research Synthesis Methods. 2020;n/a(n/a).
